# Efficacy and safety of lenvatinib versus sorafenib in first-line treatment of advanced hepatocellular carcinoma: A meta-analysis

**DOI:** 10.3389/fonc.2022.1010726

**Published:** 2022-12-22

**Authors:** Jia Luo, Benjian Gao, Zhiyu Lin, Hua Fan, Wen Ma, Danfei Yu, Qian Yang, Jing Tian, Xiaoli Yang, Bo Li

**Affiliations:** ^1^ Department of Oncology and Hematology, People’s Hospital of Leshan, Leshan, China; ^2^ Department of General Surgery (Hepatobiliary Surgery), The Affiliated Hospital of Southwest Medical University, Luzhou, China; ^3^ Academician (Expert) Workstation of Sichuan Province, Luzhou, China

**Keywords:** hepatocellular carcinoma, lenvatinib, sorafenib, systemic therapy, meta-analysis

## Abstract

**Objective:**

Lenvatinib and sorafenib are first-line oral multikinase inhibitors approved for the treatment of advanced hepatocellular carcinoma (HCC). However, the choice of the primary therapeutic agent among these two remains controversial. This meta-analysis aimed to estimate the efficacy and safety of lenvatinib and sorafenib in patients with advanced HCC.

**Methods:**

PubMed, Cochrane Library, Web of Science, and Embase databases were searched for relevant research published up to June 30, 2022. After quality assessment and data extraction of the included studies, RevMan 5.3 software was used for analysis. Odds ratio (OR) and hazard ratio (HR) with a 95% confidence interval (CI) were calculated using a fixed-effects or random-effects model.

**Results:**

Fifteen studies containing 3908 patients were included after final scrutiny. Our meta-analysis showed that there was no significant difference in overall survival (OS) between the lenvatinib and sorafenib groups (HR = 0.86; 95% CI: 0.72–1.02; *p* = 0.09); however, the progression-free survival (PFS) (HR = 0.63; 95% CI: 0.53–0.74; *p* < 0.00001), complete response (CR) (OR = 5.61; 95% CI: 2.71–11.64; *p* < 0.00001), partial response (PR) (OR = 4.62; 95% CI: 3.06–6.98; *p* < 0.00001), objective response rate (ORR) (OR = 5.61; 95% CI: 3.90–8.09; *p* < 0.00001), and disease control rate (DCR) (OR = 2.42; 95% CI: 1.79–3.28; *p* < 0.00001) in the lenvatinib group were significantly better than those in the sorafenib group. In terms of treatment safety, lenvatinib had similar incidences of any grade adverse events (AEs) (OR = 0.99; 95% CI: 0.47–2.09; *p* = 0.98) and grade ≥ 3 AEs (OR = 1.17, 95% CI; 1.00–1.37; *p* = 0.05) compared to sorafenib. Besides, lenvatinib was significantly associated with a higher incidence of hypertension, proteinuria, fatigue, decreased appetite, and weight loss, whereas sorafenib was associated with a higher incidence of diarrhea and hand-foot skin reaction (*p* < 0.05).

**Conclusion:**

Given its potential survival benefit and good tolerability, lenvatinib is an appropriate and promising alternative to sorafenib as first-line systemic therapy in patients with advanced HCC.

**Systematic review registration:**

https://www.crd.york.ac.uk/prospero/, identifier: CRD 42022327398.

## 1 Introduction

Hepatocellular carcinoma (HCC), the most common type of primary liver cancer, ranks as the fourth leading cause of cancer-associated deaths worldwide (1). For patients with early-stage HCC, curative treatments such as surgical resection, transplantation, and ablation, have been shown to improve survival (2, 3). However, HCC is generally diagnosed at an advanced stage and usually occurs in people with chronic liver disease, limiting the feasibility of such curative therapies. For patients with advanced HCC, systemic therapy is the primary treatment option which is shown to significantly improve the overall survival (OS) and quality of life of HCC patients (4).

Sorafenib is an oral multikinase inhibitor that modulates multiple tumor-signaling pathways by inhibiting several receptor tyrosine kinases, such as vascular endothelial growth factor receptor (VEGFR) 1-3, platelet-derived growth factor receptor (PDGFR), KIT, and RET; and downstream Raf signaling molecules (5–7). A phase 3 randomized controlled trial (RCT) which enrolled 601 patients with advanced HCC revealed that the median OS was significantly improved with sorafenib treatment compared to the placebo group (10.7 months vs. 7.9 months, hazard ratio [HR]: 0.69; 95% confidence interval [CI]: 0.55–0.87, *p* < 0.001) (8). Further, the outcome of another phase 3 RCT involving patients from the Asia-Pacific region indicated a similar observation that sorafenib treatment improved the OS (6.5 months in sorafenib vs. 4.2 months in placebo, HR: 0.68; 95% CI: 0.50–0.93, *p* < 0.014) (9). Since then, several multikinase inhibitors have been developed, but none of them have shown non-inferiority or superiority to sorafenib as a first-line therapy for advanced HCC (10–12).

In 2018, the REFLECT trial demonstrated that lenvatinib, an oral multikinase inhibitor, was non-inferior to sorafenib in terms of OS for the treatment of advanced HCC (median OS: 13.6 months for lenvatinib vs. 12.3 months for sorafenib, HR: 0.92; 95% CI: 0.79–1.06) (13, 14). In addition, lenvatinib showed a significant improvement in progression-free survival (PFS) and objective response rate (ORR). Owing to these encouraging results, lenvatinib became the second therapeutic agent approved for first-line systemic treatment for advanced HCC. Although several subsequent studies have been conducted to compare the efficacy of lenvatinib and sorafenib, they have yielded inconsistent results. Hence, for the treatment of patients with advanced HCC, the choice of the primary systemic therapeutic agent remains controversial. In this meta-analysis, we comprehensively evaluated the clinical efficacy and safety of lenvatinib, thereby providing a more reliable basis for clinical decision-making.

## 2 Materials and methods

### 2.1 Protocol and registration

This review was performed in compliance with the guidelines of the Preferred Reporting Items for Systematic Reviews and Meta-Analyses (PRISMA) Statement (15). Besides, the prospective protocol for this study was registered with the PROSPERO (Registration number: CRD 42022327398).

### 2.2 Search strategy

All studies evaluating the efficacy of lenvatinib and sorafenib on advanced HCC were identified by searching PubMed, Cochrane Library, Web of Science, and Embase databases from inception until June 30, 2022. The search keywords or the medical subject headings (MeSH) terms were as follows: “hepatocellular carcinoma”, “liver cell carcinoma”, “liver cancer”, “hepatoma”, “lenvatinib”, and “sorafenib”. The search strategy used in PubMed was as follows: ((((hepatocellular OR hepato‐cellular OR hepatic OR liver) and (carcinom* OR cancer OR neoplasm* OR malign* OR tumor)) OR hepatocellular carcinoma OR HCC) OR “Carcinoma, Hepatocellular”[MeSH] OR Liver Neoplasms[MeSH]) AND (((((((sorafenib) OR (Nexavar)) OR (BAY 43-9006)) OR (Sorafenib N-Oxide)) OR (BAY-673472)) OR (BAY 545-9085)) OR (Sorafenib Tosylate)) OR (“Sorafenib”[Mesh]) AND (((((((((lenvatinib) OR (Lenvima)) OR (E 7080)) OR (ER-203492-00)) OR (E-7080 mesylate)) OR (lenvatinib metabolite M2)) OR (lenvatinib mesylate)) OR (lenvatinib methanesulfonate)) OR (lenvatinib mesilate)) OR (“lenvatinib” [Supplementary Concept]). Furthermore, the reference lists of the included studies or the relevant reviews were checked manually to identify other potentially eligible studies. The literature search was limited to articles written in English language.

### 2.3 Inclusion and exclusion criteria

Two authors independently screened the results of initial searches, and any disagreement was resolved *via* discussion with a third author. The inclusion criteria were as follows (1): all prospective or retrospective studies comparing the efficacy of lenvatinib with sorafenib in the treatment of advanced HCC; (2) all trial participants with histologically or radiologically diagnosed advanced HCC, who were not previously treated with systemic therapies; (3) experimental intervention: lenvatinib; (4) control intervention: sorafenib; and (5) studies reporting at least one of the following outcomes: OS, PFS, ORR, disease control rate (DCR), complete response (CR), partial response (PR), and adverse events (AEs). The exclusion criteria were as follows: (1) studies without a control group; (2) case reports, abstracts, letters, reviews, conference reports, or expert opinions; and (3) studies without the full text. In the case of replication studies based on the same study patients, we included the most comprehensive and up-to-date data.

### 2.4 Data extraction

Three authors reviewed the full text of the eligible studies and extracted data independently. Any discrepancies or disagreements in the extracted data were solved through consensus in a plenum. Data extraction was performed using a single form that included the following items: the first author, date of publication, region, study type, sample size, drug dose, the main condition of patients, and outcome indicators. The hazard ratios of time-to-event variables (OS and PFS) were extracted directly from the original studies or estimated indirectly through the reported number of events and the relevant *p* value for the log-rank statistics.

### 2.5 Quality assessment

The Cochrane risk of bias assessment tool (16) was used to evaluate the quality of the selected RCTs based on the following seven items: random sequence generation, allocation concealment, blinding of participants and personnel, blinding of outcome and assessment, incomplete outcome data, selective reporting, and other bias. Each item was graded as high, low, or unclear risk of bias. In addition, the quality of the included non-randomized comparative studies was assessed using the Newcastle-Ottawa scale (NOS) (17). This scale measures quality based on three parameters: selection, comparability, and outcome assessment, with a maximum of 9 points. Studies with a score of more than 6 were determined to be of high quality.

### 2.6 Statistical analysis

Statistical analyses were performed using the Cochrane Review Manager software (RevMan, version 5.3). The primary endpoints in this meta-analysis were OS and DFS, and the effect sizes were determined by HR with 95% CI. Dichotomous variables were assessed by OR with 95% CI. Besides, between-study heterogeneity was evaluated using the *χ^2^* test and expressed by the *I^2^* index. Heterogeneity was regarded as significant when the *p* < 0.1 or *I^2^* > 50%. The random-effects model was used to calculate the pooled data if heterogeneity was significant; otherwise, the fixed-effects model was adopted. Potential publication bias was assessed by visually inspecting the funnel plots. Sensitivity analysis was conducted by removing each study in turn. A *p* < 0.05 was considered statistically significant.

## 3 Results

### 3.1 Literature search

**Figure 1:**
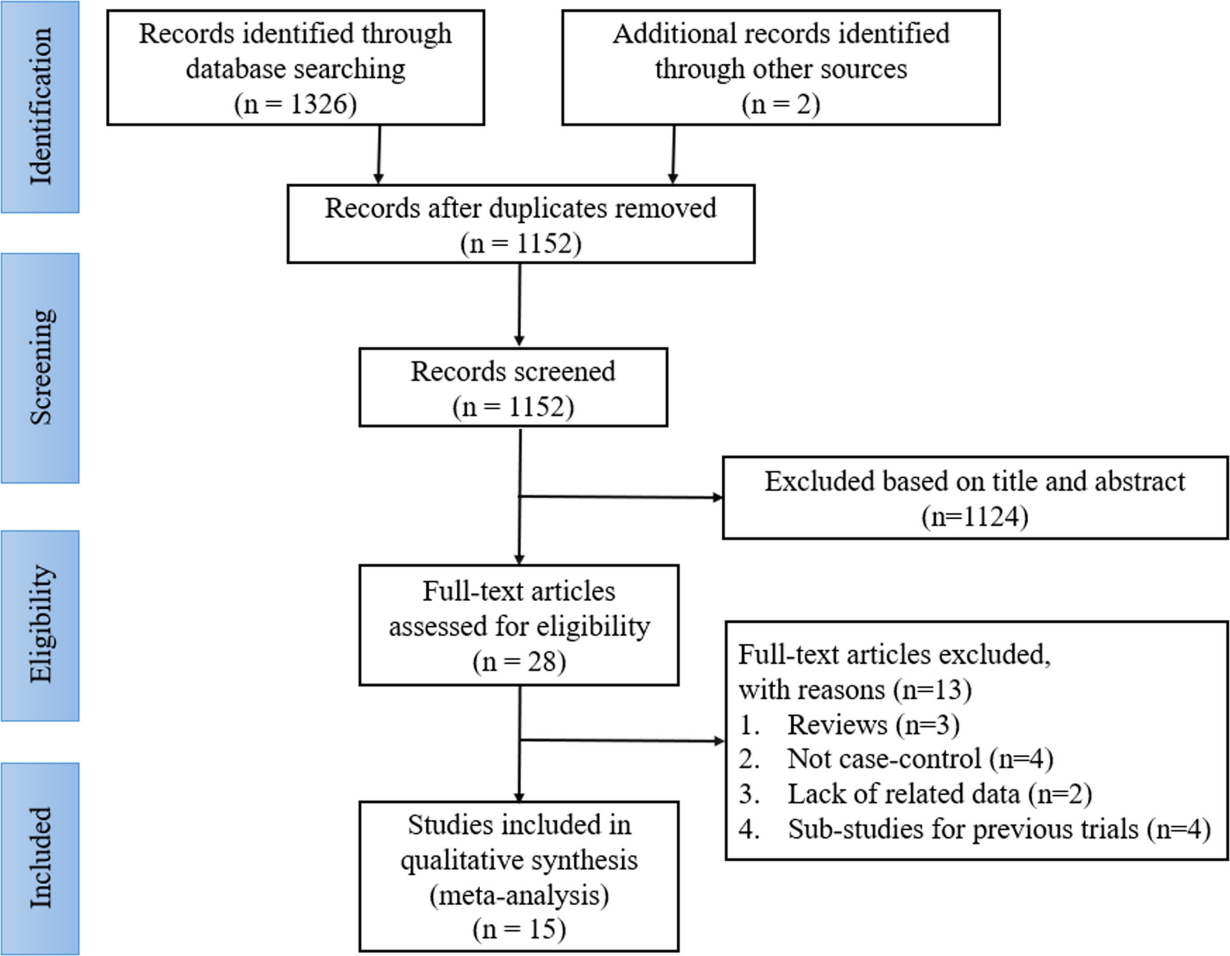
Flowchart of the study selection process.

A total of 1328 records were identified through the initial search; of which, 176 articles were removed for duplication, and 1124 studies were discarded after scanning the titles and abstracts. After a detailed reading and full text assessment, 13 articles were further excluded as they did not meet the inclusion criteria as 3 of them were reviews, 4 were not case-control studies, 2 lacked the related data, and 4 were sub-studies of previous trials. Finally, 15 articles were included in this analysis, including 1 RCT (13) and 14 retrospective cohort studies (RCS) (18–31). The literature selection process is shown in **Figure 1**.

### 3.2 Study characteristics and quality assessment

**Figure 2:**
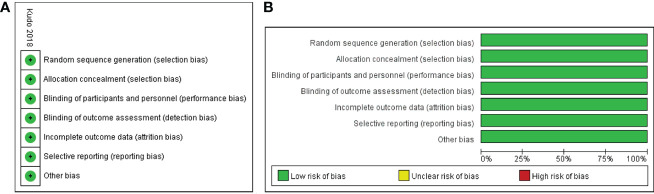
Assessment of risk of bias for RCT. Risk of bias summary **(A)**; risk of bias graph **(B)**.

All eligible studies included a total of 3908 participants: 1722 in the lenvatinib group and 2186 in the sorafenib group. The published year ranged from 2018 to 2022, and the regions studied included Asia, Europe, and North America. The dosage of the drugs was consistent in the majority of the studies (13, 18, 19, 21–24, 26–28, 31). For instance, the initial dose of sorafenib was 400 mg twice daily, while lenvatinib was administered at a dose of 12 mg once daily for patients with body weights ≥ 60 kg or 8 mg once daily for those with body weights < 60 kg. The characteristics of the included studies are summarized in **Table 1**. The bias risk of one RCT (13) was assessed using the Cochrane Collaboration tool and determined to be low (**Figure 2**). Besides, the 14 retrospective studies (18–31) had NOS scores ranging from 7 to 9, indicating a high quality of data in all included studies (**Table 1**).

**Table 1:** Characteristics of the included studies.

Author (year)	Region	Study type	Intervention	Sample size	Age (Years)	Gender (M/F)	BCLC stage: B/C	Child-Pugh class: A/B	ECOG score: 0/1	NOS
Kudo (13) (2018)	Asia, European, North American	RCT	Lenv	478	63.0 (20–88)	405/73	104/374	475/3	304/174	–
			Sora	476	62.0 (22–88)	401/75	92/384	471/5	301/175	
Kuzuya (18) (2020)	Japan	RCS	Lenv	13	70.0 (53–92)	11/2	0/13	13/0	12/1	7
			Sora	28	67.0 (35–82)	21/7	0/28	28/0	18/10	
Lee (19) (2020)	Korea	RCS	Lenv	43	60 (32–85)	35/8	8/35	37/6	16/27	7
			Sora	55	63 (43–86)	42/13	8/47	52/3	22/33	
Nakano (20) (2020)	Japan	RCS	Lenv	146	72.8 ± 9.6	125/21	79/67	134/12	NA	9
			Sora	146	72.8 ± 8.5	121/25	81/65	137/9		
Terashima (21) (2020)	Japan	RCS	Lenv	45	Median:70	33/12	NA	39/6	36/8	7
			Sora	135	Median:69	96/39	NA	114/21	106/22	
Burgio (22) (2020)	Italy	RCS	Lenv	144	< 70: 52.8%	111/33	36/108	137/7	114/30	7
			Sora	144	< 70: 52.7%	119/25	36/108	134/10	114/30	
Casadei (23) (2020)	Italy, Japan and Korea	RCS	Lenv	385	72.1 ± 10.0	303/82	NA/175	339/46	NA	8
			Sora	555	62.6 ± 11.5	485/70	NA/483	512/43	NA	
Fukushima (24) (2021)	Japan	RCS	Lenv	110	73.0 (67.3–78.0)	91/19	59/49	86/24	NA	7
			Sora	110	72.0 (67.0–78.0)	94/16	47/62	85/25	NA	
Kim (25) (2021)	Korea	RCS	Lenv	44	56.0 (51.0–66.3)	39/5	NA	36/8	41/3	8
			Sora	61	64.0 (58.0–70.5)	51/10	NA	56/5	59/2	
Kuo (26) (2021)	China	RCS	Lenv	70	65.0 ± 12.3	50/20	14/56	68/2	NA	8
			Sora	140	65.7 ± 11.6	100/40	25/115	138/2	NA	
Rimini (27) (2021)	Italy and Japan	RCS	Lenv	92	< 65: 25%	75/17	36/56	87/5	70/22	8
			Sora	92	< 65: 35.87%	81/11	36/56	85/7	65/27	
Tomonari (28) (2021)	Japan	RCS	Lenv	52	70 (53–88)	36/16	27/25	52/0	38/14	8
			Sora	52	71 (43–85)	35/17	29/23	52/0	37/15	
Choi (29) (2022)	Korea	RCS	Lenv	44	58 (51.5–64.8)	40/4	4/39	29/13	32/12	7
			Sora	88	58 (52.3–64.8)	80/8	8/77	63/19	55/33	
Lee (30) (2022)	China	RCS	Lenv	22	63.95 ± 11.38	18/4	0/22	22/0	NA	8
			Sora	44	63.77 ± 10.53	36/8	0/44	44/0	NA	
Park (31) (2022)	Korea	RCS	Lenv	34	62 (55–67)	29/5	1/29	0/30	NA	7
			Sora	60	65 (56–72)	52/8	4/52	0/56	NA	

NA, not available; RCT, randomized controlled trial; RCS, retrospective cohort study; Lenv, lenvatinib; Sora, sorafenib; M, male; F, female; BCLC, Barcelona clinic liver cancer; NOS, Newcastle-Ottawa scale.

### 3.3 Efficacy analysis

#### 3.3.1 OS

**Figure 3:**
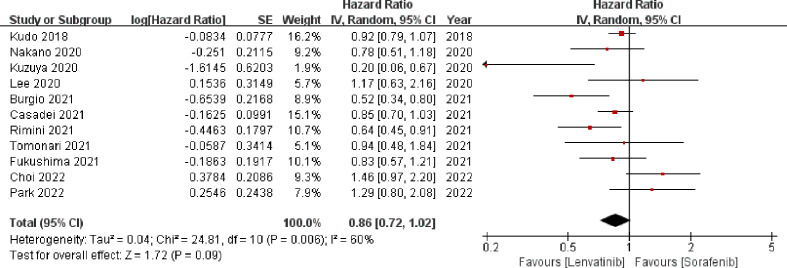
Forest plot on OS. OS, overall survival.

Eleven studies (13, 18–20, 22–24, 27–29, 31) involving 3347 patients reported OS. The meta-analysis indicated that there was no significant difference in the OS between the two groups (HR = 0.86; 95% CI: 0.72–1.02; *p* = 0.09). A random-effects model was used, as statistical heterogeneity was identified among the included studies (*p* = 0.006, *I^2^* = 60%; **Figure 3**). On the contrary, the pooled analysis showed that OS was significantly higher in the lenvatinib group as compared to the sorafenib group (HR = 0.90; 95% CI: 0.82–1.00; *p* = 0.04) when the heterogeneity was reduced (*p* = 0.12, *I^2^* = 38%) by excluding two trials (18, 22).

#### 3.3.2 PFS

**Figure 4:**
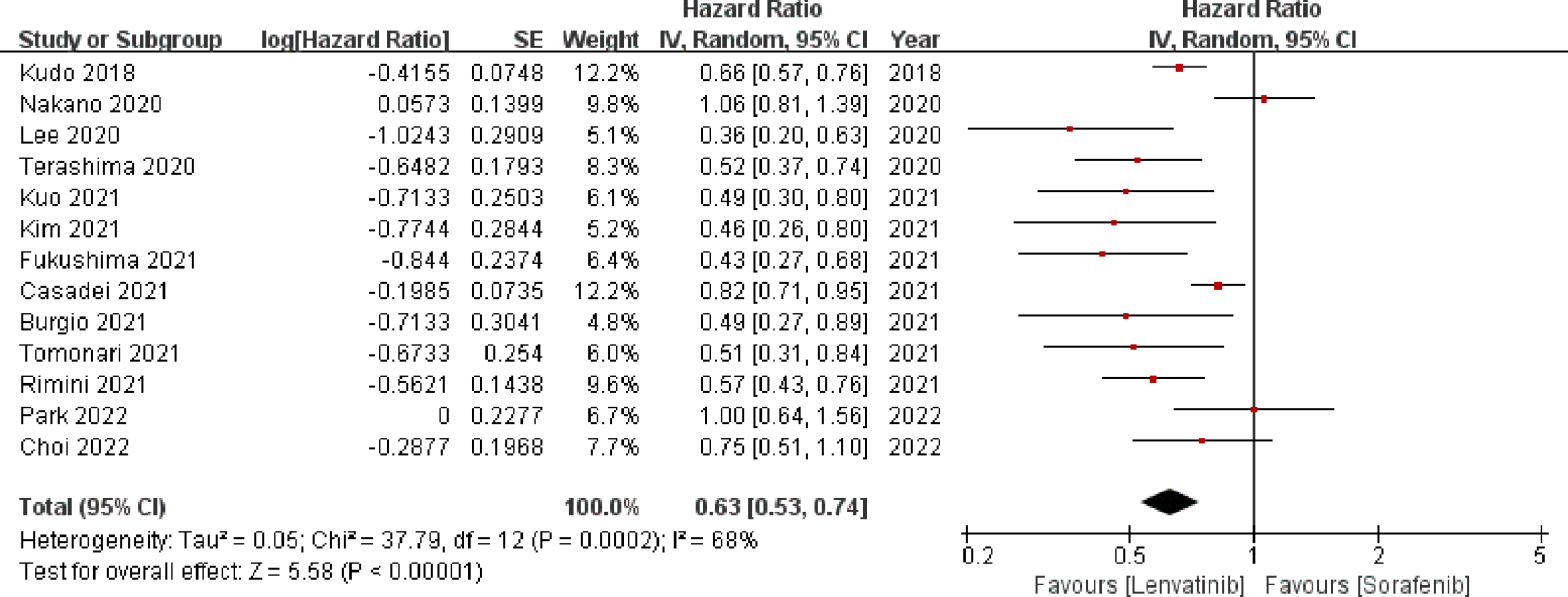
Forest plot on PFS. PFS, progression-free survival.

Thirteen studies (13, 19–29, 31) enrolling 3760 patients provided data concerning PFS. The pooled analysis showed that compared with sorafenib, lenvatinib was associated with significantly improved PFS (HR = 0.63; 95% CI: 0.53–0.74; *p* < 0.00001). A random-effects model was used, due to statistical heterogeneity (*p* = 0.0002, *I^2^* = 68%; **Figure 4**). To reduce the heterogeneity, two studies (20, 23) were removed (*p* = 0.10, *I^2^* = 38%). The recalculated results consistently showed that the treatment with lenvatinib was associated with greater improvement in PFS compared with sorafenib (HR = 0.60; 95% CI: 0.55–0.67; *p* < 0.00001).

#### 3.3.3 Treatment response

**Figure 5:**
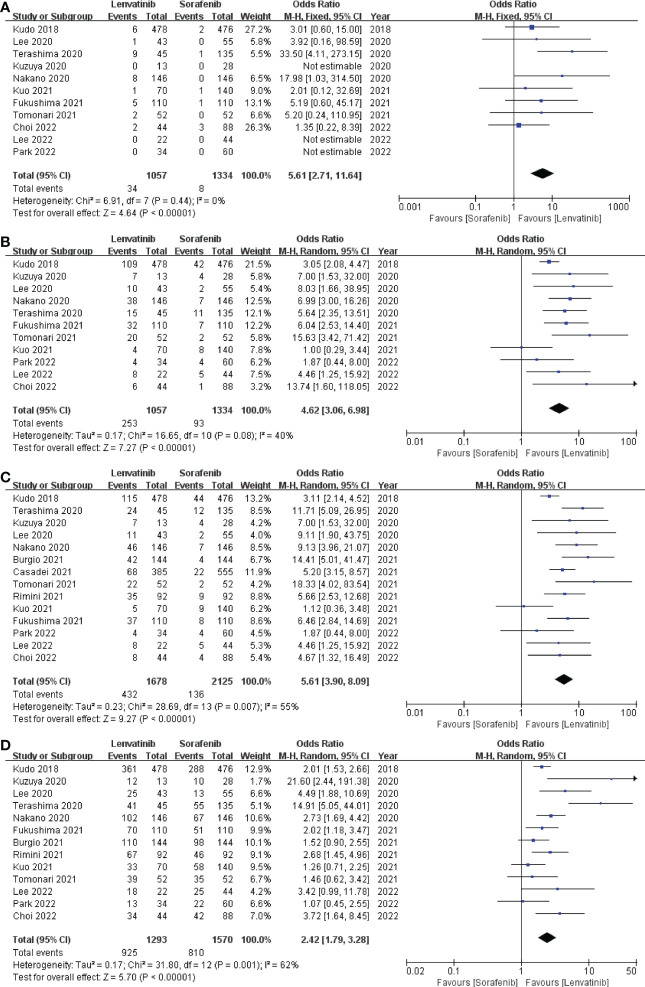
Forest plot on CR **(A)**, PR **(B)**, ORR **(C)**, and DCR **(D)**. CR, complete response; PR, partial response; ORR, objective response rate; DCR, disease control rate.

In this study, CR, PR, ORR, and DCR were used to evaluate tumor treatment response. Eleven studies (13, 18–21, 24, 26, 28–31) which included 2391 patients reported CR and PR, fourteen studies (13, 18–24, 26–31) which enrolled 3803 patients investigated ORR, and thirteen studies (13, 18–22, 24, 26–31) which recruited 2863 patients documented DCR. The pooled analysis showed that CR (3.22% vs. 0.60%; OR = 5.61; 95% CI: 2.71–11.64; *p* < 0.00001; **Figure 5A**), PR (23.94% vs. 6.97%; OR = 4.62; 95% CI: 3.06–6.98; *p* < 0.00001; **Figure 5B**), ORR (25.74% vs. 6.4%; OR = 5.61; 95% CI: 3.90–8.09; *p* < 0.00001; **Figure 5C**), and DCR (71.54% vs. 51.59%; OR = 2.42; 95% CI: 1.79–3.28; *p* < 0.00001; **Figure 5D**) of the lenvatinib group were better than those of the sorafenib group.

### 3.4 Safety analysis

**Figure 6:**
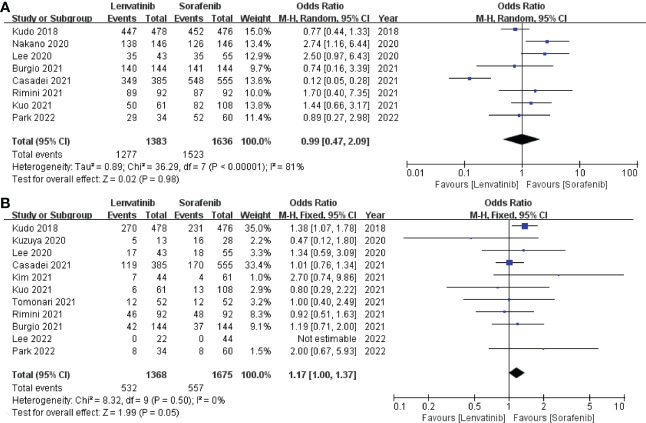
Forest plot on any grade AEs **(A)** and grade ≥ 3 AEs **(B)**. AEs, adverse events.

The incidence of any grade AEs was reported in 8 studies (13, 19, 20, 22, 23, 26, 27, 31), which included a total of 3019 patients. The pooled analysis showed no significant difference in the incidence of any grade AEs between the lenvatinib group (92.34%) and the sorafenib group (93.09%) (OR = 0.99; 95% CI: 0.47–2.09; *p* = 0.98; **Figure 6A**). The incidence of grade ≥ 3 AEs was reported in 11 studies (13, 18, 19, 22, 23, 25–28, 30, 31), which involved a total of 3043 patients. Similarly, the pooled data indicated no significant difference in the incidence of grade ≥ 3 AEs between the two groups, with lenvatinib and sorafenib groups exhibiting 38.89% and 33.25%, respectively (OR = 1.17; 95% CI: 1.00–1.37; *p* = 0.05; **Figure 6B**).

Treatment of HCC with tyrosine kinase inhibitors (TKIs) could lead to some common AEs, including hand-foot skin reaction, diarrhea, hypertension, decreased appetite, decreased weight, fatigue, and proteinuria. The pooled analysis showed that the incidence of hand-foot skin reaction and diarrhea was significantly lower in the lenvatinib group compared to the sorafenib group. Whereas, the incidence of hypertension, decreased appetite, weight loss, fatigue, and proteinuria in the lenvatinib group was significantly higher than in the sorafenib group (**Table 2**).

**Table 2:** Comparison of the incidence of common AEs between the two groups.

Outcomes	No. of studies	Incidence rate (%)		Heterogeneity		The pooled analysis		
		Lenvatinib	Sorafenib	*I^2^*	*p*	OR	95% CI	*p*
Hand-foot skin reaction	12 (13, 18–20, 22, 23, 25–28, 30, 31)	23.58	43.68	38%	0.09	0.39	0.33–0.45	< 0.00001
Diarrhea	11 (13, 18, 19, 22, 23, 25–28, 30, 31)	27.56	35.04	47%	0.04	0.67	0.50–0.90	0.007
Decreased appetite	9 (13, 18–20, 25–28, 31)	33.02	21.61	71%	0.0006	1.87	1.13–3.11	0.02
Weight loss	2 (13, 25)	29.12	20.11	4%	0.31	1.59	1.20–2.12	0.01
Hypertension	12 (13, 18–20, 22, 23, 25–28, 30, 31)	36.21	24.22	71%	< 0.0001	2.65	1.78–3.93	< 0.00001
Fatigue	9 (13, 20, 22, 25–28, 30, 31)	33.55	22.15	75%	< 0.0001	1.78	1.12–2.83	0.02
Proteinuria	8 (13, 18–20, 25, 26, 28, 31)	18.94	7.00	0	0.46	3.07	2.27–4.15	< 0.00001

AEs, adverse events; OR, odds ratio; CI, confidence interval.

### 3.5 Subgroup analysis

Subgroup analyses were further performed based on the study design and region, yielded similar results to the primary analysis except for the incidence of any grade AEs; the subgroup of Asian region showed the incidence of any grade AEs was significantly lower in the sorafenib group compared to the lenvatinib group (OR = 1.86; 95% CI: 1.18–2.92; *p* = 0.008). The results are summarized in **Table 3**.

**Table 3:** Results of subgroup analyses.

Outcomes	No. of studies	No. of Patients	Heterogeneity		The pooled analysis		
			*I^2^*	*p*	HR/OR	95% CI	*p*
RCS							
OS	10 (18–20, 22–24, 27–29, 31)	2393	63%	0.004	0.84	0.67–1.05	0.13
PFS	12 (19–29, 31)	2806	70%	0.0001	0.62	0.51–0.75	< 0.00001
CR	10 (18–21, 24, 26, 28–31)	1437	8%	0.37	6.59	2.89–15.00	< 0.00001
PR	10 (18–21, 24, 26, 28–31)	1437	29%	0.18	5.18	3.27–8.19	< 0.00001
ORR	13 (18–24, 26–31)	2849	41%	0.06	6.15	4.27–8.87	< 0.00001
DCR	12 (18–22, 24, 26–31)	1909	65%	0.0009	2.54	1.76–3.67	< 0.00001
Any grade AEs	7 (19, 20, 22, 23, 26, 27, 31)	2065	83%	< 0.00001	1.04	0.41–2.67	0.93
Grade ≥ 3 AEs	10 (18, 19, 22, 23, 25–28, 30, 31)	2089	0	0.66	1.06	0.87–1.30	0.55
Asian region							
OS	7 (18–20, 24, 28, 29, 31)	981	51%	0.03	0.96	0.71–1.30	0.80
PFS	9 (19–21, 24–26, 28, 29, 31)	1394	72%	0.0004	0.60	0.46–0.78	0.0002
CR	10 (18–21, 24, 26, 28–31)	1437	8%	0.37	6.59	2.89–15.00	< 0.00001
PR	10 (18–21, 24, 26, 28–31)	1437	29%	0.18	5.18	3.27–8.19	< 0.00001
ORR	10 (18–21, 24, 26, 28–31)	1437	48%	0.05	5.80	3.50–9.61	< 0.00001
DCR	10 (18–21, 24, 26, 28–31)	1437	69%	0.0007	2.77	1.76–4.37	< 0.0001
Any grade AEs	4 (19, 20, 26, 31)	653	0	0.39	1.86	1.18–2.92	0.008
Grade ≥ 3 AEs	7 (18, 19, 25, 26, 28, 30, 31)	677	1%	0.41	1.18	0.78–1.79	0.43

HR, hazard ratio; OR, odds ratio; CI, confidence interval; RCS, retrospective cohort study; OS, overall survival; PFS, progression-free survival; CR, complete response; PR, partial response; ORR, objective response rate; DCR, disease control rate; AEs, adverse events.

### 3.6 Publication bias

**Figure 7:**
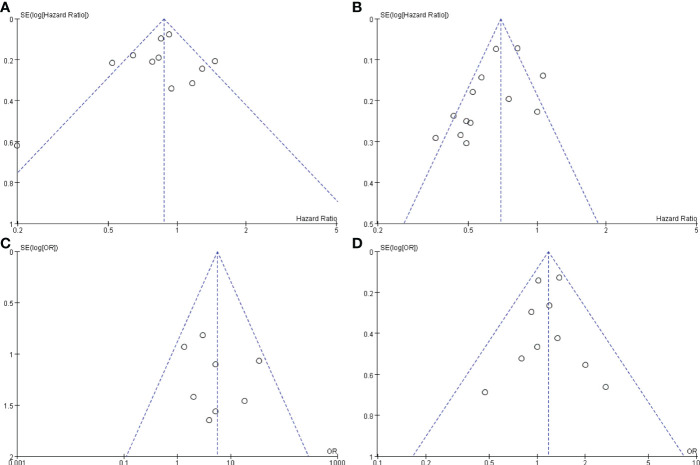
Funnel plots based on OS **(A)**, PFS **(B)**, CR **(C)**, and grade ≥3 AEs **(D)**. OS, overall survival; PFS, progression-free survival; CR, complete response; AEs, adverse events.

To understand whether there is any publication bias influencing our study, funnel plots were drawn for OS, PFS, CR, and grade ≥3 AEs. The funnel plots of the studies were not asymmetrical and were evenly vertically distributed, demonstrating no or limited publication bias (**Figure 7**).

## 4 Discussion

Being one of the most prevalent malignant tumors, HCC poses a major threat to human health. Due to its insidious onset, most patients are diagnosed at an advanced stage and are not eligible for curative treatments. Therefore, systemic therapy plays a crucial role in the treatment of advanced HCC, and the TKIs sorafenib and lenvatinib are currently the most effective first-line monotherapies (32).

Lenvatinib is a selective, multi-targeted TKI of VEGFR1-3 and other receptor tyrosine kinases associated with proangiogenic and oncogenic pathways, including FGFR1-4, PDGFRα, cKIT, and RET (33, 34). Compared to sorafenib, the distinguishing features of lenvatinib is its potent activity against FGFR1-4 (35). Besides, recent studies have revealed that lenvatinib has immunomodulatory activity (36–38). Preliminary data from a clinical trial have also shown that the therapeutic combination of lenvatinib with pembrolizumab resulted in an ORR of 46%, exhibiting promising efficacy in advanced HCC (39). Furthermore, the cost-utility analysis showed that lenvatinib offered similar clinical effectiveness at a lower cost than sorafenib, indicating that lenvatinib may be a cost-saving alternative in patients with advanced HCC (40). However, recent studies that compared the efficacy of lenvatinib and sorafenib in HCC found conflicting results (18–31, 41), and hence the optimal choice for the patient between these two drugs remains controversial. Therefore, our primary aim to perform this systematic review is to evaluate the feasibility and safety of lenvatinib as a first-line treatment for advanced HCC.

In the present study, our findings suggested that there was no significant difference in the OS between the lenvatinib and sorafenib groups. However, the lenvatinib group demonstrated a significantly better outcome in terms of OS than the sorafenib group after the heterogeneity was reduced by excluding outlier trials. Besides, we also found that the PFS, CR, PR, ORR, and DCR values in the lenvatinib group were significantly superior to those in the sorafenib group, indicating the therapeutic advantage of lenvatinib. These results were generally consistent with the results of most of the included studies, in which lenvatinib was non-inferior to sorafenib in terms of OS. A multicentric analysis of 184 patients with advanced HCC in Italy and Japan reported the median OS being 15.2 and 10.5 months for lenvatinib and sorafenib arms, first demonstrating the superiority of lenvatinib over sorafenib regarding the OS in a real-world setting (27). Similarly, recent real-world data from 466 patients in Italy showed a significant advantage in the OS for lenvatinib compared to sorafenib as first-line therapy for advanced HCC (22). Notably, the subgroup analyses showed that patients with objective response had significantly better median OS than those with progressive disease in both sorafenib and lenvatinib groups (18, 20, 30). Although OS is an unbiased primary endpoint for evaluating novel agents in oncology investigations, it has been suggested that PFS and ORR might be better surrogate endpoints. Both PFS and ORR reliably reflect survival benefits and could be assessed before the administration of additional efficacious drugs (42). Besides, Llovet et al. (43) confirmed that PFS had a significant correlation with OS at the trial level and that PFS with a threshold of HR ≤0.6 was highly predictive of a significant improvement in OS. This could explain the significant difference in PFS between the two groups in our study, with HR reaching 0.63 (95% CI: 0.53–0.74), while there was no significant difference in OS.

Regarding treatment safety, this meta-analysis found that lenvatinib had similar incidences of any grade AEs (92.34% vs. 93.09%) and grade ≥ 3 AEs (38.89% vs. 33.25%) compared to sorafenib. Even though the incidence was comparable, lenvatinib and sorafenib showed significant differences in the type of AEs. For instance, lenvatinib was associated with a higher incidence of hypertension, proteinuria, fatigue, decreased appetite, and weight loss, whereas sorafenib was associated with a higher incidence of diarrhea and hand-foot skin reaction. Considering the balance between safety and efficacy and to minimize early dose reduction or interruption, the recommended starting dose of lenvatinib was 8 mg per day for patients weighing < 60 and 12 mg per day for patients weighing ≥ 60 kg (44, 45). The safety profiles of lenvatinib and sorafenib in this study were consistent with those observed in previous studies, which further confirmed that lenvatinib was well tolerated as first-line therapy for advanced HCC.

Similar results were reported in a previous meta-analysis conducted by Facciorusso et al. (41) which included 5 studies involving a total of 1481 patients. The authors compared the efficacy of lenvatinib and sorafenib as first-line therapy for advanced HCC. Their study showed that there was no significant difference in the outcome of OS between the two groups (HR = 0.81; 95% CI: 0.58–1.11); however, lenvatinib significantly improved PFS (HR = 0.67; 95% CI: 0.48–0.94), ORR (OR = 7.70; 95% CI: 2.99–19.82), and DCR (OR = 2.41; 95% CI: 1.55–3.77) compared to sorafenib. Besides, the incidence of severe AEs in the lenvatinib group was 64.9%, which was comparable to that in the sorafenib group (56.4%; OR = 1.31; 95% CI: 0.82–2.09). These results indicated that lenvatinib is associated with a longer PFS and higher response rates as compared to sorafenib, revealing a significantly better therapeutic effect. However, in contrast to our study, the analysis of Facciorusso et al. (41) included only 5 studies with relatively small sample sizes, which might affect the reliability of the results. In addition, our study also conducted a comprehensive comparative analysis of common AEs to confirm the good tolerability of lenvatinib.

Nonetheless, our study has several limitations. First, significant heterogeneity among studies in some outcomes was observed, which could be attributed to parameters such as different study designs, population demographics, follow-up times, and interventions. Second, our analysis was limited by studies published in English language, and therefore omission of relevant articles published in other languages is a possibility. Finally, most of the included studies (n=14) were retrospective and nonrandomized, suggesting that unmeasured confounders and selection or recall bias may have influenced the results of these studies.

## 5 Conclusion

This systematic review and meta-analysis showed that lenvatinib potentially has a survival advantage over sorafenib in terms of OS, in addition to having significant gains in PFS, CR, PR, ORR, and DCR. Moreover, the safety profiles of lenvatinib and sorafenib were found to be similar and well-tolerated. In conclusion, our study shows that lenvatinib is an appropriate and promising first-line systemic therapy for advanced HCC. However, given the limitations of this analysis, further large-sample and high-quality RCTs are required to conclusively establish this finding in the future.

## Data availability statement

The original contributions presented in the study are included in the article/supplementary material. Further inquiries can be directed to the corresponding authors.

## Author contributions

JL, BG, and BL conceived and designed the study. ZL and HF screened electronic databases. WM, DY, and QY extracted data from the selected articles. JT and XY evaluated eligible study quality and potential bias risk. Statistical analyses were performed by BG. JL and BG wrote the manuscript. JL and BL supervised the study. All authors contributed to the article and approved the submitted version.
